# Supplementary material for: Mental health services in response to the COVID-19 pandemic in high-income countries: protocol for a rapid review
Source: BMJ Open. 2022 Jun 30;12(6):e062078. doi: 10.1136/bmjopen-2022-062078 (PMC9247322; doi:10.1136/bmjopen-2022-062078)
Supplement: Author's manuscript [file bmjopen-2022-062078.draft_revisions.pdf]

BMJ Open is committed to open peer review. As part of this commitment we make the peer review history of every article we publish publicly available.

When an article is published we post the peer reviewers' comments and the authors' responses online. We also post the versions of the paper that were used during peer review. These are the versions that the peer review comments apply to.

The versions of the paper that follow are the versions that were submitted during the peer review process. They are not the versions of record or the final published versions. They should not be cited or distributed as the published version of this manuscript.

BMJ Open is an open access journal and the full, final, typeset and author-corrected version of record of the manuscript is available on our site with no access controls, subscription charges or pay-per-view fees (http://bmjopen.bmj.com).

If you have any questions on BMJ Open's open peer review process please email info.bmjopen@bmj.com

BMJ Open

The impact of changes in mental health services in response to COVID-19 on people with mental health conditions: protocol for a rapid review

Journal:	*BMJ Open*
Manuscript ID	bmjopen-2022-062078
Article Type:	Protocol
Date Submitted by the Author:	16-Feb-2022

Complete List of Authors:	Yu, Ge; Newcastle University, Population Health Sciences Institute<br>Craig, Dawn; Institute of Health & Society, Newcastle University, Health Economics Group, Institute of Health and Society<br>Fu, Yu; Newcastle University Faculty of Medical Sciences, Population Health Sciences Institute

Keywords:	MENTAL HEALTH, COVID-19, Adult psychiatry < PSYCHIATRY, Protocols & guidelines < HEALTH SERVICES ADMINISTRATION & MANAGEMENT

SCHOLARONE™ Manuscripts

The impact of changes in mental health services in response to COVID-19 on people with mental health conditions: protocol for a rapid review

G Yu*[1,2], D Craig[1,2], Y Fu[1,2]

1. NIHR Applied Research Collaborative North East and North Cumbria, Cumbria, Northumberland and Tyne and Wear NHS Foundation Trust, The Clocktower Building, St Nicholas Hospital, Gosforth, Newcastle NE3 3XT, UK
2. Population Health Sciences Institute, Faculty of Medical Sciences, Newcastle University, Sir James Spence Building, Royal Victoria Infirmary, Newcastle upon Tyne NE1 4LP, UK

*Correspondence

Dr Ge Yu

Baddiley-Clark Building
Richardson Road
Population Health Sciences Institute
Newcastle University
Newcastle
NE2 4AX

ge.yu@ncl.ac.uk

01912 085307

Keywords: mental health, mental health conditions, psychotic disorders, mental health service, COVID-19, rapid review

Word count: 1780

For peer review only - http://bmjopen.bmj.com/site/about/guidelines.xhtml

For peer review only

ABSTRACT

Introduction

The COVID-19 pandemic has caused disruptions to mental health services, forcing the rapid implementation of alternative ways of delivering services alongside a greater immediate, and continuously growing, demand across those services. The care and level of mental health service provided are felt to be inadequate to respond to the increasing demand for mental health conditions in the time of the pandemic, leading to an urgent need to learn from service change and consequences to inform solutions and plans to support the NHS post-pandemic plan in the UK. This rapid review aims to understand the changes in mental health services during the pandemic and summarise the impact of these changes on the health outcomes of people with mental health conditions.

Methods and analysis

Cochrane CENTRAL, MEDLINE, EMBASE, and PsycInfowill be searched for eligible studies with key terms indicating mental health AND COVID-19 AND health services. Studies will be included if objective and subjective effects of changes to mental health services in response to COVID-19 are reported on adults with mental health conditions, peer-reviewed and published in the English language. Study selection and data extraction will be undertaken dependently by two reviewers. Evidence will be summarised narratively and in a logic model.

Ethics and dissemination

Ethics approval is not required for this review. A list of interventions/services/models of care delivered to people with mental health conditions will be grouped as “Do”, “Don’t” and “Don’t know” based on the evidence on effectiveness and acceptability. The results will be written for publication in an open-access peer-reviewed journal and disseminated to the public and patients, clinicians, commissioners, funders, and academic conferences.

PROSPERO registration number

CRD42022306923

For peer review only

For peer review only - http://bmjopen.bmj.com/site/about/guidelines.xhtml

Strengths and limitations of this study

- This is a rapid review with a systematic search of literature
- Evidence on changes to mental health services and associated health outcomes will be examined to make practice recommendations
- This review will provide the evidence base to inform solutions and plans to support the mental health service provision for the post-pandemic period
- Some limitations to the study design include limited to OECD studies, exclusion of non-English studies, publication bias, quality of data, selection bias, and no quality assessment in the rapid evidence review.

For peer review only

For peer review only - http://bmjopen.bmj.com/site/about/guidelines.xhtml

INTRODUCTION

In the UK, up to December 2021, 1 in 7 people have had a positive COVID-19 test result. It is recognised that as well as the physical effects, a COVID-19 diagnosis has adverse mental health effects both in previously healthy people and those with pre-existing mental health conditions.[1] About 1 in 5 people with COVID-19 have experienced poorer mental health within 90 days of diagnosis.[2] It is estimated that the pandemic will lead to new or additional mental health support for up to 10 million people in England (around 20% of the population).[3] Evidence from previous studies illustrated that the virus and periods of lockdown have deteriorated population mental health and disproportionately worsened the mental health burden for certain population subgroups including those with pre-existing mental health conditions.[4 5] People with existing psychiatric diagnoses have reported increased symptoms and poorer access to services and support leading to relapse and suicidal behaviour.[1] At the same time, they are more likely to contract COVID-19 than people with no history of poor mental health. The adaptations required to enable the delivery of mental healthcare during this period of extended infection-control measures could have been disproportionally detrimental to those now living with mental health conditions that have arisen since the first UK-wide lockdown began in March 2020. Difficulties attending review appointments in person and closure of support services are likely to have impacted all those in, or in need of, active treatment.[6] The unequal impact of the pandemic is likely to further entrench and exacerbate the existing structural inequalities in mental health among people with pre-existing mental health conditions before COVID-19, and the mental health services provided have failed to meet their increasing demand for mental health conditions during the time of the COVID-19 pandemic.[7]

The NHS has set up a long term plan to improve mental healthcare services that are widely regarded as being under-resourced.[8] However, for people with mental health conditions, there is an incomplete picture of the impact of the pandemic on the pattern of mental health services. Despite bringing current service inadequacies to the forefront, the pandemic could provide an opportunity to rethink conventional approaches to mental health services planning to meet patients' needs. For example, remote community treatment and support has long been suggested but has not previously been implemented widely because of barriers and challenges from both healthcare staff and service users. Since the onset of the pandemic, the situation has changed.[9] Similarly, the threshold for hospital admission for mental illness varies between individuals and requires continuous adaptation over time. Therefore, learning from health service changes throughout the pandemic, and their consequences for people's physical and mental health is vital to inform practical policy solutions for integrated service recovery and effectively plan services that reach those with the greatest need.

The WHO recommends rapid review methods as an efficient approach to provide rapid but relevant and contextualised evidence to the health decision-makers when there are time, resources or other logistical constraints.[10-15] We will conduct a rapid review to synthesise the evidence on changes in mental health services because of the COVID-19 pandemic and their impacts in high-income countries.[16 17]

For peer review only - http://bmjopen.bmj.com/site/about/guidelines.xhtml

METHODS AND ANALYSIS

A rapid review will be undertaken to provide an evidence base supporting the recommendation of mental health services and identify areas where the evidence base is lacking, and future research is required. The review will be guided by the Cochrane guidance for rapid reviews [18]. Preferred Reporting Items for Systematic reviews and Meta-Analyses extension for Rapid Reviews guidance[19] will be followed for reporting.

This protocol has been developed in advance of the review to improve the transparency and quality of the methods to help reduce bias and enhance the reproducibility of the results. This has been registered with the PROSPECT CRD42022306923.

Eligibility

Type of studies

Peer-reviewed quantitative or qualitative empirical studies describing the setting, problems addressed, resource requirements, aim, service components, provider, method of delivery, objective and subjective effects of changes to mental health services in response to COVID-19 will be included. Included studies will be those undertaken in an Organisation for Economic Co-operation and Development (OECD) country[20], to ensure a degree of commonality in the health system and socioeconomic and demographic context.

Type of participants

People aged 18 or over experiencing mental health conditions as described by NHS[21] who were in need of mental health support during the pandemic.

Type of health services

Interventions, services and models of care delivered in response to COVID-19 to provide support for adults with mental health conditions will be included.

Type of outcome measures

Primary outcomes are objective measures and subjective effects of changes, efficacy or use of a service by mental health patients. Secondary outcomes are changes in knowledge, attitudes or satisfaction of service users and/or professionals and health inequalities.

Search methods

Cochrane CENTRAL, MEDLINE, Embase and PsycInfowill be searched for from 2019 to the present. A search strategy has been developed for MEDLINE with support from an independent information specialist, using a range of keywords and subject headings representing COVID-19, mental health and low- and middle-income countries (Appendix). This will be used to inform the detailed search strategy for other databases. Reference lists and citation indexes of relevant studies will also be examined. Only OECD studies published in or after 2019 and in the English language (no resource available for translation) will be searched.

Selection of studies

Studies identified from databases will be exported to EndNote X9[22] for deduplication. Study titles of abstracts will be screened independently according to the selection criteria. Any results that are inconclusive at the initial screen will be included and considered at full-text

For peer review only

screening. All full-text papers will be screened independently by two researchers (GY and YF). Any discrepancies will be resolved by discussion and consensus. Where there is a disagreement between two reviewers, a third researcher (DC) will be consulted to reach a consensus.

Data extraction

A data extraction sheet will be designed to capture information including author's first name, publication date, setting, study design, sample size, mental health conditions, characteristics of participants, service components, service provider, method of delivery, resources required, outcome measures and main study results. GY will extract all the data. YF will check for accuracy and completeness through random double-extraction of 10% of included studies. Where a study appears to have multiple citations, original authors will be contacted for clarification. All information from multiple citations will be used if no replies are received.

Quality assessment and quality control

This is a rapid review, so no quality assessment will be conducted. This can be done retrospectively if time and resources allow.

The following steps will be taken to ensure quality control for the searching, screening, data extraction, and coding process. GY will conduct screening and data extraction following pre-determined inclusion criteria and data extraction framework. For articles that are retrieved and full text saved, YF will check 10% of the coding to ensure they meet the screening criteria. Where there is a disagreement between two reviewers, a third researcher (DC) will be consulted to reach a consensus. Synthesis of each outcome will be conducted by GY and independently revised by YF.

Data synthesis and analysis

A tabulated and narrative synthesis of the results will be undertaken following current best practice[23-25] to conduct synthesis systematically and transparently. It will focus on the mental health services, mechanisms and their impact on health outcomes. A logic model will be produced to present context, service provision and outcomes. Possible unintended adverse outcomes will also be reported. Also, a list of interventions/services/models of care delivered to people with mental health conditions will be grouped as "Do", "Don't" and "Don't know" based on the strength of the evidence on effectiveness and acceptability.

If data is available, outcomes of studies will be synthesised according to characteristics of study participants, for example, deprived communities, ethnic minorities, to produce evidence on health inequalities that is likely to have been exacerbated during the pandemic.

PATIENT AND PUBLIC INVOLVEMENT

This study has been designed and developed in consultation with two public members (one with lived experience), to ensure their input on the study design. They both read and commented on the review summary, search strategies, eligibility and plans to synthesise data and dissemination strategies. They valued the potential impact of this review on NHS plans for mental health post-pandemic. It has been agreed that the process of this rapid review will be presented to both members for their further comments.

For peer review only - http://bmjopen.bmj.com/site/about/guidelines.xhtml

ETHICS AND DISSEMINATION

As this rapid review will only consider published literature, no ethics approval is needed. Dissemination will be led by the research team and supported by the public member and the wider project advisory group. Results of this review will contribute to reports which will be produced and shared with the National Institute for Health Research (NIHR)Three Research Schools and NIHR Applied Research Collaboration (ARC) North East and North Cumbria (NENC). The findings will be published in peer-reviewed journals and a plain study summary will be disseminated to people receiving mental health care, groups and forum that the project public members are connected, practitioners and commissioners. An abstract will be prepared for academic conferences such as the Society for Academic Primary Care Annual Conference.

CONCLUSION

This rapid review protocol proposes methods for rapid evidence synthesis. A list of interventions/services/models of care delivered to people with mental health problems during the pandemic will be grouped as “Do”, “Don’t” and “Don’t know” based on the strength of the evidence on effectiveness and acceptability. The proposed rapid review is expected to have an immediate impact on the care of patients by increasing awareness of the impacts associated with service changes and factors enabling high-quality services, in both affected individuals and their service providers. Evidence and practise recommendations produced will be useful in supporting the NHS strategy for planning mental health services in a post-pandemic future, and also in highlighting the needs of service users, potentially the needs of underserved populations. In addition, it will identify important knowledge gaps to help guide research direction.

Accepting the potentially increased risk of biases introduced by rapid review methods is a compromise for many rapid review protocols.[26] The decision to restrict the evidence review to OECD studies, research published in English and a limited number of databases, and not to undertake a formal quality appraisal exercise will increase the risk of bias and the likelihood that relevant research may be missed or excluded. To help mitigate the limitations of the proposed study design, strategies such as regular team meetings throughout and publishing the protocol in advance of conducting the review have been employed. Furthermore, a quality appraisal may be undertaken retrospectively at a later date for a more comprehensive assessment of the evidence.

For peer review only

For peer review only - http://bmjopen.bmj.com/site/about/guidelines.xhtml

AUTHORS' CONTRIBUTIONS

GY and YF conceived the study idea and design. GY drafted the initial manuscript, and all authors reviewed the manuscript and provided input to the final version.

FUNDING STATEMENT

This project/research/work is supported by the National Institute of Health Research (NIHR) Three Research Schools (award number: MHF018) and the NIHR Applied Research Collaboration (ARC) for the North East and North Cumbria (NENC) (award number N/A). The NIHR is the nation's largest funder of health and care research and provides the people, facilities and technology that enables research to thrive. Find out more about the NIHR.

COMPETING INTERESTS STATEMENT

The authors declare that they have no competing interests.

For peer review only

For peer review only - http://bmjopen.bmj.com/site/about/guidelines.xhtml

REFERENCES

1. Moreno C, Wykes T, Galderisi S, et al. How mental health care should change as a consequence of the COVID-19 pandemic. *The Lancet Psychiatry* 2020;7(9):813-24. doi: 10.1016/S2215-0366(20)30307-2
2. Rogers JP, Chesney E, Oliver D, et al. Psychiatric and neuropsychiatric presentations associated with severe coronavirus infections: a systematic review and meta-analysis with comparison to the COVID-19 pandemic. *The Lancet Psychiatry* 2020;7(7):611-27. doi: https://doi.org/10.1016/S2215-0366(20)30203-0
3. O'Shea N. Covid-19 and the nation's mental health: Forecasting needs and risks in the UK. 2020
4. Pierce M, Hope H, Ford T, et al. Mental health before and during the COVID-19 pandemic: a longitudinal probability sample survey of the UK population. *The Lancet Psychiatry* 2020;7(10):883-92.
5. Iob E, Steptoe A, Fancourt D. Abuse, self-harm and suicidal ideation in the UK during the COVID-19 pandemic. *The British Journal of Psychiatry* 2020;217(4):543-46.
6. Anderson G, Frank JW, Naylor CD, et al. Using socioeconomics to counter health disparities arising from the covid-19 pandemic. *Bmj* 2020;369
7. Bambra C, Riordan R, Ford J, et al. The COVID-19 pandemic and health inequalities. *J Epidemiol Community Health* 2020;74(11):964-68.
8. Alderwick H, Dixon J. The NHS long term plan: British Medical Journal Publishing Group, 2019.
9. Shore JH, Schneck CD, Mishkind MC. Telepsychiatry and the coronavirus disease 2019 pandemic—current and future outcomes of the rapid virtualization of psychiatric care. *JAMA psychiatry* 2020;77(12):1211-12.
10. Tricco AC, Langlois E, Straus SE, et al. Rapid reviews to strengthen health policy and systems: a practical guide: World Health Organization 2017.
11. McIntosh HM, Calvert J, Macpherson KJ, et al. The healthcare improvement Scotland evidence note rapid review process: providing timely, reliable evidence to inform imperative decisions on healthcare. *JBI Evidence Implementation* 2016;14(2):95-101.
12. Tricco AC, Antony J, Zarin W, et al. A scoping review of rapid review methods. *BMC medicine* 2015;13(1):1-15.
13. Polisena J, Garritty C, Kamel C, et al. Rapid review programs to support health care and policy decision making: a descriptive analysis of processes and methods. *Systematic reviews* 2015;4(1):1-7.
14. Featherstone RM, Dryden DM, Foisy M, et al. Advancing knowledge of rapid reviews: an analysis of results, conclusions and recommendations from published review articles examining rapid reviews. *Systematic reviews* 2015;4(1):1-8.
15. Grant MJ, Booth A. A typology of reviews: an analysis of 14 review types and associated methodologies. *Health information & libraries journal* 2009;26(2):91-108.
16. Brunier A, Drysdale C. COVID-19 disrupting mental health services in most countries, WHO survey. *World Heal Organ* 2020
17. Byrne A, Barber R, Lim CH. Impact of the COVID-19 pandemic–a mental health service perspective. *Progress in Neurology and Psychiatry* 2021;25(2):27-33b.
18. Garritty C, Gartlehner G, Kamel C, et al. Cochrane Rapid Reviews Interim Guidance from the Cochrane Rapid Reviews Methods Group. March 2020, 2020.
19. Page MJ, McKenzie JE, Bossuyt PM, et al. Updating guidance for reporting systematic reviews: development of the PRISMA 2020 statement. *Journal of Clinical Epidemiology* 2021;134:103-12.
20. Reibling N, Ariaans M, Wendt C. Worlds of Healthcare: A Healthcare System Typology of OECD Countries. *Health Policy* 2019;123(7):611-20. doi: https://doi.org/10.1016/j.healthpol.2019.05.001
21. NHS. Mental health conditions: NHS; 2022 [Available from: https://www.nhs.uk/mental-health/conditions/ accessed 08 Feb 2022.

For peer review only

For peer review only - http://bmjopen.bmj.com/site/about/guidelines.xhtml

22. EndNote [program]. EndNote X9 version. Philadelphia, PA: Clarivate, 2013.
23. Campbell M, Katikireddi SV, Sowden A, et al. Improving Conduct and Reporting of Narrative Synthesis of Quantitative Data (ICONS-Quant): protocol for a mixed methods study to develop a reporting guideline. *BMJ open* 2018;8(2):e020064.
24. Popay J, Roberts H, Sowden A, et al. Guidance on the conduct of narrative synthesis in systematic reviews. *A product from the ESRC methods programme Version* 2006;1:b92.
25. Campbell M, McKenzie JE, Sowden A, et al. Synthesis without meta-analysis (SWiM) in systematic reviews: reporting guideline. *bmj* 2020;368
26. Ganann R, Ciliska D, Thomas H. Expediting systematic reviews: methods and implications of rapid reviews. *Implementation Science* 2010;5(1):1-10.

For peer review only

For peer review only - http://bmjopen.bmj.com/site/about/guidelines.xhtml

The impact of changes in mental health services in response to COVID-19 on people with mental health conditions: protocol for a rapid review

Appendix: Search Strategy

PubMed/Medline

	**PubMed/Ovid Medline (1950 to date)**
**COVID-19**	(2019 nCoV[tiab] OR 2019nCoV[tiab] OR corona virus[tiab] OR corona viruses[tiab] OR coronavirus[tiab] OR coronaviruses[tiab] OR COVID[tiab] OR COVID19[tiab] OR nCov 2019[tiab] OR SARS-CoV2[tiab] OR SARS CoV-2[tiab] OR SARSCoV2[tiab] OR SARSCoV-2[tiab] OR "COVID-19"[Mesh] OR "COVID-19 Testing"[Mesh] OR "COVID-19 Vaccines"[Mesh] OR "Coronavirus"[Mesh:NoExp] OR "Receptors, Coronavirus"[Mesh] OR "SARS-CoV-2"[Mesh] OR "Spike Glycoprotein, Coronavirus"[Mesh]) NOT ("animals"[mh] NOT "humans"[mh]) NOT (editorial[pt] OR newspaper article[pt]
**MENTAL HEALTH**	1. EATING DISORDERS/ or ANOREXIA NERVOSA/ or BINGE-EATING DISORDER/ or BULIMIA NERVOSA/ or FEMALE ATHLETE TRIAD SYNDROME/ or PICA/<br>2. HYPERPHAGIA/ or BULIMIA/<br>3. SELF-INJURIOUS BEHAVIOR/ or SELF MUTILATION/ or SUICIDE/ or SUICIDAL IDEATION/ or SUICIDE, ATTEMPTED/<br>4. MOOD DISORDERS/ or AFFECTIVE DISORDERS, PSYCHOTIC/ or BIPOLAR DISORDER/ or CYCLOTHYMIC DISORDER/ or DEPRESSIVE DISORDER/ or DEPRESSION, POSTPARTUM/ or DEPRESSIVE DISORDER, MAJOR/ or DEPRESSIVE DISORDER, TREATMENT-RESISTANT/ or DYSTHYMIC DISORDER/ or SEASONAL AFFECTIVE DISORDER/<br>5. NEUROTIC DISORDERS/<br>6. DEPRESSION/<br>7. ADJUSTMENT DISORDERS/<br>8. exp ANTIDEPRESSIVE AGENTS/<br>9. ANXIETY DISORDERS/ or AGORAPHOBIA/ or NEUROCIRCULATORY ASTHENIA/ or OBSESSIVE-COMPULSIVE DISORDER/ or OBSESSIVE HOARDING/ or PANIC DISORDER/ or PHOBIC DISORDERS/ or STRESS DISORDERS, TRAUMATIC/ or COMBAT DISORDERS/ or STRESS DISORDERS, POST-TRAUMATIC/ or STRESS DISORDERS, TRAUMATIC, ACUTE/<br>10. ANXIETY/ or ANXIETY, CASTRATION/ or KORO/<br>11. ANXIETY, SEPARATION/<br>12. PANIC/<br>13. exp ANTI-ANXIETY AGENTS/<br>14. SOMATOFORM DISORDERS/ or BODY DYSMORPHIC DISORDERS/ or CONVERSION DISORDER/ or HYPOCHONDRIASIS/ or NEURASTHENIA/<br>15. HYSTERIA/<br>16. MUNCHAUSEN SYNDROME BY PROXY/ or MUNCHAUSEN SYNDROME/<br>17. FATIGUE SYNDROME, CHRONIC/<br>18. OBSESSIVE BEHAVIOR/<br>19. COMPULSIVE BEHAVIOR/ or BEHAVIOR, ADDICTIVE/

For peer review only

	20. IMPULSE CONTROL DISORDERS/ or FIRESETTING BEHAVIOR/ or GAMBLING/ or TRICHOTILLOMANIA/<br>21. STRESS, PSYCHOLOGICAL/ or BURNOUT, PROFESSIONAL/<br>22. SEXUAL DYSFUNCTIONS, PSYCHOLOGICAL/ or VAGINISMUS/<br>23. ANHEDONIA/<br>24. AFFECTIVE SYMPTOMS/<br>25. *MENTAL DISORDERS/<br>26. (eating disorder* or anorexia nervosa or bulimi* or binge eat* or (self adj (injur* or mutilat*)) or suicide* or suicidal or parasuicid* or mood disorder* or affective disorder* or bipolar i or bipolar ii or (bipolar and (affective or disorder*)) or mania or manic or cyclothymic* or depression or depressive or dysthymi* or neurotic or neurosis or adjustment disorder* or antidepress* or anxiety disorder* or agoraphobia or obsess* or compulsi* or panic or phobi* or ptsd or posttrauma* or post trauma* or combat or somatoform or somati#ation or medical* unexplained or body dysmorphi* or conversion disorder or hypochondria* or neurastheni* or hysteria or munchausen or chronic fatigue* or gambling or trichotillomania or vaginismus or anhedoni* or affective symptoms or mental disorder* or mental health).ti.<br>27. or/1-26
**LOW AND MIDDLE INCOME COUNTRIES**	(afghanistan OR albania OR algeria OR american samoa OR angola OR "antigua and barbuda" OR antigua OR barbuda OR argentina OR armenia OR armenian OR aruba OR azerbaijan OR bahrain OR bangladesh OR barbados OR republic of belarus OR belarus OR byelarus OR belORussia OR byelORussian OR belize OR british honduras OR benin OR dahomey OR bhutan OR bolivia OR "bosnia and herzegovina" OR bosnia OR herzegovina OR botswana OR bechuanaland OR brazil OR brasil OR bulgaria OR burkina faso OR burkina fasso OR upper volta OR burundi OR urundi OR cabo verde OR cape verde OR cambodia OR kampuchea OR khmer republic OR cameroon OR cameron OR cameroun OR central african republic OR ubangi shari OR chad OR chile OR china OR colombia OR comORos OR comORo islands OR iles comORes OR mayotte OR democratic republic of the congo OR democratic republic congo OR congo OR zaire OR costa rica OR "cote d'ivoire" OR "cote d' ivoire" OR cote divoire OR cote d ivoire OR ivORy coast OR croatia OR cuba OR cyprus OR czech republic OR czechoslovakia OR djibouti OR french somaliland OR dominica OR dominican republic OR ecuadOR OR egypt OR united arab republic OR el salvadOR OR equatORial guinea OR spanish guinea OR eritrea OR estonia OR eswatini OR swaziland OR ethiopia OR fiji OR gabon OR gabonese republic OR gambia OR "geORgia (republic)" OR geORgian OR ghana OR gold coast OR gibraltar OR greece OR grenada OR guam OR guatemala OR guinea OR guinea bissau OR guyana OR british guiana OR haiti OR hispaniola OR honduras OR hungary OR india OR indonesia OR timOR OR iran OR iraq OR isle of man OR jamaica OR jORdan OR kazakhstan OR kazakh OR kenya OR "democratic people's republic of kORea" OR republic of kORea OR nORth kORea OR south kORea OR kORea OR kosovo OR kyrgyzstan OR kirghizia OR kirgizstan OR kyrgyz republic OR kirghiz OR laos OR lao pdr

For peer review only

	OR "lao people's democratic republic" OR latvia OR lebanon OR lebanese republic OR lesotho OR basutoland OR liberia OR libya OR libyan arab jamahiriya OR lithuania OR macau OR macao OR republic of nORth macedonia OR macedonia OR madagascar OR malagasy republic OR malawi OR nyasaland OR malaysia OR malay federation OR malaya federation OR maldives OR indian ocean islands OR indian ocean OR mali OR malta OR micronesia OR federated states of micronesia OR kiribati OR marshall islands OR nauru OR nORthern mariana islands OR palau OR tuvalu OR mauritania OR mauritius OR mexico OR moldova OR moldovian OR mongolia OR montenegro OR mORocco OR ifni OR mozambique OR pORtuguese east africa OR myanmar OR burma OR namibia OR nepal OR netherlands antilles OR nicaragua OR niger OR nigeria OR oman OR muscat OR pakistan OR panama OR papua new guinea OR new guinea OR paraguay OR peru OR philippines OR philipines OR phillipines OR phillippines OR poland OR "polish people's republic" OR pORtugal OR pORtuguese republic OR puerto rico OR romania OR russia OR russian federation OR ussr OR soviet union OR union of soviet socialist republics OR rwanda OR ruanda OR samoa OR pacific islands OR polynesia OR samoan islands OR navigatOR island OR navigatOR islands OR "sao tome and principe" OR saudi arabia OR senegal OR serbia OR seychelles OR sierra leone OR slovakia OR slovak republic OR slovenia OR melanesia OR solomon island OR solomon islands OR nORfolk island OR nORfolk islands OR somalia OR south africa OR south sudan OR sri lanka OR ceylon OR "saint kitts and nevis" OR "st. kitts and nevis" OR saint lucia OR "st. lucia" OR "saint vincent and the grenadines" OR saint vincent OR "st. vincent" OR grenadines OR sudan OR suriname OR surinam OR dutch guiana OR netherlands guiana OR syria OR syrian arab republic OR tajikistan OR tadjikistan OR tadzhikistan OR tadzhik OR tanzania OR tanganyika OR thailand OR siam OR timOR leste OR east timOR OR togo OR togolese republic OR tonga OR "trinidad and tobago" OR trinidad OR tobago OR tunisia OR turkey OR turkmenistan OR turkmen OR uganda OR ukraine OR uruguay OR uzbekistan OR uzbek OR vanuatu OR new hebrides OR venezuela OR vietnam OR viet nam OR middle east OR west bank OR gaza OR palestine OR yemen OR yugoslavia OR zambia OR zimbabwe OR nORthern rhodesia OR global south OR africa south of the sahara OR sub-saharan africa OR subsaharan africa OR africa, central OR central africa OR africa, nORthern OR nORth africa OR nORthern africa OR magreb OR maghrib OR sahara OR africa, southern OR southern africa OR africa, eastern OR east africa OR eastern africa OR africa, western OR west africa OR western africa OR west indies OR indian ocean islands OR caribbean OR central america OR latin america OR "south and central america" OR south america OR asia, central OR central asia OR asia, nORthern OR nORth asia OR nORthern asia OR asia, southeastern OR southeastern asia OR south eastern asia OR southeast asia OR south east asia OR asia, western OR western asia OR europe, eastern OR east europe OR eastern europe OR developing country OR developing countries OR developing nation? OR developing population? OR developing wORld OR less developed countr* OR less developed nation? OR less developed population? OR less developed wORld OR

For peer review only

	lesser developed countr* OR lesser developed nation? OR lesser developed population? OR lesser developed wORld OR under developed countr* OR under developed nation? OR under developed population? OR under developed wORld OR underdeveloped countr* OR underdeveloped nation? OR underdeveloped population? OR underdeveloped wORld OR middle income countr* OR middle income nation? OR middle income population? OR low income countr* OR low income nation? OR low income population? OR lower income countr* OR lower income nation? OR lower income population? OR underserved countr* OR underserved nation? OR underserved population? OR underserved wORld OR under served countr* OR under served nation? OR under served population? OR under served wORld OR deprived countr* OR deprived nation? OR deprived population? OR deprived wORld OR poOR countr* OR poOR nation? OR poOR population? OR poOR wORld OR poORer countr* OR poORer nation? OR poORer population? OR poORer wORld OR developing econom* OR less developed econom* OR lesser developed econom* OR under developed econom* OR underdeveloped econom* OR middle income econom* OR low income econom* OR lower income econom* OR low gdp OR low gnp OR low gross domestic OR low gross national OR lower gdp OR lower gnp OR lower gross domestic OR lower gross national OR lmic OR lmics OR third wORld OR lami countr* OR transitional countr* OR emerging economies OR emerging nation?).ti,ab,sh,kf.
**Notes**	[pt=publication type; ab=abstract; ti=title; fs=floating subheading; sh=subject heading; mp=title, original title, abstract, name of substance word, subject heading word, unique identifier]

For peer review only

PRISMA-P (Preferred Reporting Items for Systematic review and Meta-Analysis Protocols) 2015 checklist: recommended items to address in a systematic review protocol*

Section and topic	Item No	Checklist item	Line and Page number(s)
**ADMINISTRATIVE INFORMATION**			
Title:			
Identification	1a	Identify the report as a protocol of a systematic review	Page 1
Update	1b	If the protocol is for an update of a previous systematic review, identify as such	NA
Registration	2	If registered, provide the name of the registry (such as PROSPERO) and registration number	Page 5
Authors:			
Contact	3a	Provide name, institutional affiliation, e-mail address of all protocol authors; provide physical mailing address of corresponding author	Page 1
Contributions	3b	Describe contributions of protocol authors and identify the guarantor of the review	Page 8
Amendments	4	If the protocol represents an amendment of a previously completed or published protocol, identify as such and list changes; otherwise, state plan for documenting important protocol amendments	NA
Support:			
Sources	5a	Indicate sources of financial or other support for the review	Page 8
Sponsor	5b	Provide name for the review funder and/or sponsor	Page 8
Role of sponsor or funder	5c	Describe roles of funder(s), sponsor(s), and/or institution(s), if any, in developing the protocol	NA
**INTRODUCTION**			
Rationale	6	Describe the rationale for the review in the context of what is already known	Page 4
Objectives	7	Provide an explicit statement of the question(s) the review will address with reference to participants, interventions, comparators, and outcomes (PICO)	NA
**METHODS**			
Eligibility criteria	8	Specify the study characteristics (such as PICO, study design, setting, time frame) and report characteristics (such as years considered, language, publication status) to be used as criteria for eligibility for the review	Page 5
Information sources	9	Describe all intended information sources (such as electronic databases, contact with study authors, trial registers or other grey literature sources) with planned dates of coverage	Page 5
Search strategy	10	Present draft of search strategy to be used for at least one electronic database, including planned limits, such that it could be	Supplementary material

For peer review only

		repeated	
Study records:			
Data management	11a	Describe the mechanism(s) that will be used to manage records and data throughout the review	Page 5
Selection process	11b	State the process that will be used for selecting studies (such as two independent reviewers) through each phase of the review (that is, screening, eligibility and inclusion in meta-analysis)	Page 5
Data collection process	11c	Describe planned method of extracting data from reports (such as piloting forms, done independently, in duplicate), any processes for obtaining and confirming data from investigators	Page 6
Data items	12	List and define all variables for which data will be sought (such as PICO items, funding sources), any pre-planned data assumptions and simplifications	Page 6
Outcomes and prioritization	13	List and define all outcomes for which data will be sought, including prioritization of main and additional outcomes, with rationale	Page 5
Risk of bias in individual studies	14	Describe anticipated methods for assessing risk of bias of individual studies, including whether this will be done at the outcome or study level, or both; state how this information will be used in data synthesis	Page 6
Data synthesis	15a	Describe criteria under which study data will be quantitatively synthesised	Page 5
	15b	If data are appropriate for quantitative synthesis, describe planned summary measures, methods of handling data and methods of combining data from studies, including any planned exploration of consistency (such as I^2, Kendall's τ)	NA
	15c	Describe any proposed additional analyses (such as sensitivity or subgroup analyses, meta-regression)	Page 6
	15d	If quantitative synthesis is not appropriate, describe the type of summary planned	Page 6
Meta-bias(es)	16	Specify any planned assessment of meta-bias(es) (such as publication bias across studies, selective reporting within studies)	NA
Confidence in cumulative evidence	17	Describe how the strength of the body of evidence will be assessed (such as GRADE)	NA

*** It is strongly recommended that this checklist be read in conjunction with the PRISMA-P Explanation and Elaboration (cite when available) for important clarification on the items. Amendments to a review protocol should be tracked and dated.** **The copyright for PRISMA-P (including checklist) is held by the PRISMA-P Group and is distributed under a Creative Commons Attribution Licence 4.0.**

From: Shamseer L, Moher D, Clarke M, Ghersi D, Liberati A, Petticrew M, Shekelle P, Stewart L, PRISMA-P Group. Preferred reporting items for systematic review and meta-analysis protocols (PRISMA-P) 2015: elaboration and explanation. BMJ. 2015 Jan 2;349(jan02 1):g7647.

For peer review only

BMJ Open

Mental health services in response to the COVID-19 pandemic in high-income countries: protocol for a rapid review

Journal:	*BMJ Open*
Manuscript ID	bmjopen-2022-062078.R1
Article Type:	Protocol
Date Submitted by the Author:	01-May-2022
Complete List of Authors:	Yu, Ge; 1. NIHR Applied Research Collaborative North East and North Cumbria; Newcastle University, Population Health Sciences Institute<br>Craig, Dawn; Institute of Health & Society, Newcastle University, Health Economics Group, Institute of Health and Society; 1. NIHR Applied Research Collaborative North East and North Cumbria<br>Fu, Yu; 1. NIHR Applied Research Collaborative North East and North Cumbria; Newcastle University Faculty of Medical Sciences, Population Health Sciences Institute
**Primary Subject Heading**:	Mental health
Secondary Subject Heading:	Health services research, Public health
Keywords:	MENTAL HEALTH, COVID-19, Adult psychiatry < PSYCHIATRY, Protocols & guidelines < HEALTH SERVICES ADMINISTRATION & MANAGEMENT

SCHOLARONE™
Manuscripts

Mental health services in response to the COVID-19 pandemic in high-income countries: protocol for a rapid review

G Yu*[1,2], D Craig[1,2], Y Fu[1,2]

1. NIHR Applied Research Collaborative North East and North Cumbria, Cumbria, Northumberland and Tyne and Wear NHS Foundation Trust, The Clocktower Building, St Nicholas Hospital, Gosforth, Newcastle NE3 3XT, UK
2. Population Health Sciences Institute, Faculty of Medical Sciences, Newcastle University, Sir James Spence Building, Royal Victoria Infirmary, Newcastle upon Tyne NE1 4LP, UK

*Correspondence

Dr Ge Yu

Baddiley-Clark Building
Richardson Road
Population Health Sciences Institute
Newcastle University
Newcastle
NE2 4AX

ge.yu@ncl.ac.uk

01912 085307

Keywords: mental health, mental health conditions, psychotic disorders, mental health service, COVID-19, rapid review

Word count: 1722

For peer review only

For peer review only - http://bmjopen.bmj.com/site/about/guidelines.xhtml

ABSTRACT

Introduction

The COVID-19 pandemic has caused disruptions to mental health services, forcing the rapid implementation of alternative ways of delivering services alongside a greater immediate, and continuously growing, demand across those services. The care and level of mental health service provided are felt to be inadequate to respond to the increasing demand for mental health conditions in the time of the pandemic, leading to an urgent need to learn from service change and consequences to inform solutions and plans to support the NHS post-pandemic plan in the United Kingdom. This rapid review aims to understand the changes in mental health services during the pandemic and summarise the impact of these changes on the health outcomes of people with mental health conditions.

Methods and analysis

Cochrane CENTRAL, MEDLINE, EMBASE, and PsycInfowill be searched for eligible studies with key terms indicating mental health AND COVID-19 AND health services. Peer-reviewed empirical studies aiming to investigate or describe new models of care, services, initiatives or programmes developed or evolved for patients (aged 18 or over) with mental health in response to COVID, published in the English language and undertaken in a high-income country defined by Organisation for Economic Co-operation and Development (OECD) member will be included. Studies reporting views of the general public, letters of opinion to peer-review journals, editorial or commentaries will be excluded. Study selection and data extraction will be undertaken independently by two reviewers. Evidence will be summarised narratively and in a logic model.

Ethics and dissemination

Ethics approval is not required for this review. A list of interventions/services/models of care delivered to people with mental health conditions will be grouped as “Do”, “Don’t” and “Don’t know” based on the evidence on effectiveness and acceptability. The results will be written for publication in an open-access peer-reviewed journal and disseminated to the public and patients, clinicians, commissioners, funders, and academic conferences.

PROSPERO registration number

CRD42022306923

For peer review only

For peer review only - http://bmjopen.bmj.com/site/about/guidelines.xhtml

Strengths and limitations of this study

- This is a rapid review with a systematic search of literature on mental health services since the WHO declared the global outbreak of the COVID-19 pandemic on 11th March, 2022.
- This review will provide a rapid but robust collation of evidence in response to requests for timely evidence syntheses for decision-making purpose for the post-pandemic period.
- Outlining the objectives and methodology a priori will improve both transparency and quality, and help reduce bias and enhance the reproducibility of the results.
- Some limitations to the study design include studies limited to Organisation for Economic Co-operation and Development (OECD) settings, exclusion of non-English studies, publication bias, quality of data, selection bias, and no quality assessment in the rapid evidence review.
- To help mitigate the limitations of the proposed study design, studies will be screened independently by two reviewers with a third reviewer consulted when there is a lack of consensus.

For peer review only

INTRODUCTION

There was a substantial deterioration in mental health and the prevalence of mental health symptoms increased both in previously healthy people and those with pre-existing mental health conditions since the outbreak of COVID-19.[1-3] It is estimated that the pandemic will lead to new or additional mental health support for up to 10 million people in England (around 20% of the population).[4] People with existing psychiatric diagnoses have reported increased symptoms and poorer access to services and support leading to relapse and suicidal behaviour.[1] While the increasing demand for mental health support/treatment inevitably exceeded the capacity of essential mental health services, the pandemic has significantly interrupted usual practice in the United Kingdom (UK) and worldwide. On the 23 March 2020, a national lockdown was announced by the UK government with the public instructed to stay at home, socially distance and self-isolate with strict guidance about movement outside of one's household. The adaptations required to enable the delivery of mental healthcare services during this period of extended infection-control measures could have been disproportionally detrimental to those now living with mental health conditions (i.e., autism[5], obsessive compulsive disorder[6], substance use disorder[7], etc.). Difficulties attending review appointments in person and closure of support services are likely to have impacted all those in, or in need of, active treatment.[8] The unequal impact of the pandemic and country-wide lockdown is likely to further entrench and exacerbate the existing structural inequalities in mental health among people with pre-existing mental health conditions before COVID-19. Further the mental health services provided have failed to meet the increasing demand for mental health treatment/support during the time of the COVID-19 pandemic.[9]

The UK National Health Service (NHS) has set up a long term plan to improve mental healthcare services that are widely regarded as being under-resourced.[10] However, for people with mental health conditions, there is an incomplete picture of the impact of the pandemic on the pattern of mental health services. Despite bringing current service inadequacies to the forefront, the pandemic could provide an opportunity to rethink conventional approaches to mental health services planning to meet patients' needs. For example, remote community treatment and support has long been suggested but has not previously been implemented widely because of barriers and challenges from both healthcare staff and service users. Since the onset of the pandemic, the situation has changed.[11] Similarly, the threshold for hospital admission for mental illness varies between individuals and requires continuous adaptation over time. Therefore, learning from health service changes throughout the pandemic, and their consequences for people's physical and mental health is vital to inform practical policy solutions for integrated service recovery and effectively plan services that reach those with the greatest need.

THE AIM OF REVIEW

The overall aim of this review is to 1) identify changes in mental health services for adult patients in response to the pandemic, and 2) understand the impact of the changes on their health outcomes in high-income countries.

METHODS AND ANALYSIS

A rapid review is defined as "a form of knowledge synthesis that accelerates the process of conducting a traditional systematic review through streamlining or omitting various

For peer review only - http://bmjopen.bmj.com/site/about/guidelines.xhtml

methods to produce evidence for stakeholders in a resource-efficient manner".[12] The WHO recommends rapid review methods as an efficient approach to provide rapid but relevant and contextualised evidence to the health decision-makers when there are time, resources or other logistical constraints.[13-18] With rapid changes in service provision in response to the pandemic, a rapid review will be undertaken in a timely manner to provide an evidence base supporting the recommendation of mental health services since COVID-19 and identify areas where the evidence base is lacking, and future research is required. The review will be guided by the Cochrane guidance for rapid reviews[12]. Preferred Reporting Items for Systematic reviews and Meta-Analyses extension for Rapid Reviews guidance[19] will be followed for reporting. The review will be carried out between May 2022 and August 2022.

This protocol has been developed in advance of the review to improve the transparency and quality of the methods to help reduce bias and enhance the reproducibility of the results. This has been registered with the PROSPECT CRD42022306923.

Eligibility

Type of studies

Peer-reviewed quantitative, qualitative, or mixed methods empirical studies aiming to investigate or describe new models of care, services, initiatives or programmes developed or evolved for patients with mental health in response to COVID will be included. In addition, studies describing or comparing the setting, problems addressed, resource requirements, aim, service components, provider, method of delivery, objective and subjective effects of changes to mental health services in response to COVID-19 will be included. Studies reporting views of the general public, letter of opinion to peer-review journals, editorial or commentaries will be excluded.

Type of participants

People aged 18 or over experiencing mental health conditions as described by NHS[20] who were in need of mental health support during the pandemic.

Type of health services

Interventions, services and models of care delivered in response to COVID-19 to provide support for adults with mental health conditions will be included.

Type of outcome measures

Primary outcomes are objective measures and subjective effects of changes, efficacy or use of a service by mental health patients. Secondary outcomes are changes in knowledge, attitudes or satisfaction of service users and/or professionals and health inequalities.

Type of study settings

According to level of economic development of the countries or regions under study, we will use membership of the Organisation for Economic Co-operation and Development (OECD)[21] as a reference "cut-off" point to include high-income countries[22 23], to ensure a degree of similarities in the social security system, health system and socioeconomic and demographic characteristics as in the UK.

For peer review only

Search methods

Cochrane CENTRAL, MEDLINE, Embase and PsycInfowill be searched for from 2019 to the present. A search strategy has been developed for MEDLINE with support from an independent information specialist, using a range of keywords and subject headings representing COVID-19, mental health and low- and middle-income countries (see Appendix). This will be used to inform the detailed search strategy for other databases. Reference lists and citation indexes of relevant studies will also be examined. Only OECD studies published in or after 2019 and in the English language (no resource available for translation) will be searched.

Selection of studies

Studies identified from databases will be exported to EndNote X9[24] for deduplication. Study titles of abstracts will be screened independently according to the selection criteria. Any results that are inconclusive at the initial screen will be included and considered at full-text screening. All full-text papers will be screened independently by two researchers (GY and YF). Any discrepancies will be resolved by discussion and consensus. Where there is a disagreement between two reviewers, a third researcher (DC) will be consulted to reach a consensus.

Data extraction

A data extraction sheet will be designed to capture information relating to new models of care, services, initiatives or programmes developed or evolved for patients with mental health problems in response to the pandemic. Data extracted will include author's first name, publication date, setting, study design, sample size, mental health conditions, characteristics of participants, service components, service provider, method of delivery, resources required, outcome measures and main study results. GY will extract all the data. YF will check for accuracy and completeness through random double-extraction of 10% of included studies. Where a study appears to have multiple citations, original authors will be contacted for clarification. All information from multiple citations will be used if no replies are received.

Quality assessment and quality control

Quality assessment will not be conducted. Instead, a tabulated and narrative synthesis will be undertaken to report the results of included studies and discuss reasons for differences in this rapid review as suggested by current best practice guidance.[13 25 26]

The following steps will be taken to ensure quality control for the searching, screening, data extraction, and coding process. GY will conduct screening and data extraction following pre-determined inclusion criteria and data extraction framework. For articles that are retrieved and full text saved, YF will check 10% of the coding to ensure they meet the screening criteria. Where there is a disagreement between two reviewers, a third researcher (DC) will be consulted to reach a consensus. Synthesis of each outcome will be conducted by GY and independently revised by YF.

Data synthesis and analysis

A tabulated and narrative synthesis of the results will be undertaken following current best practice[25 27 28] to conduct synthesis systematically and transparently. It will focus on the

For peer review only - http://bmjopen.bmj.com/site/about/guidelines.xhtml

mental health services, mechanisms and their impact on health outcomes. A logic model will be produced to present context, service provision and outcomes. Possible unintended adverse outcomes will also be reported. Also, a list of interventions/services/models of care delivered to people with mental health conditions will be grouped as “Do”, “Don’t” and “Don’t know” based on the strength of the evidence on effectiveness and acceptability.

If data is available, outcomes of studies will be synthesised according to characteristics of study participants, for example, deprived communities, ethnic minorities, to produce evidence on health inequalities that is likely to have been exacerbated during the pandemic.

PATIENT AND PUBLIC INVOLVEMENT

This study has been designed and developed in consultation with two public members (one with lived experience), to ensure their input on the study design. They both read and commented on the review summary, search strategies, eligibility and plans to synthesise data and dissemination strategies. They valued the potential impact of this review on NHS plans for mental health post-pandemic. It has been agreed that the process of this rapid review will be presented to both members for their further comments.

ETHICS AND DISSEMINATION

As this rapid review will only consider published literature, no ethics approval is needed. Dissemination will be led by the research team and supported by the public member and the wider project advisory group. Results of this review will contribute to reports which will be produced and shared with the National Institute for Health and Care Research (NIHR)Three Research Schools and NIHR Applied Research Collaboration (ARC) North East and North Cumbria (NENC). The findings will be published in peer-reviewed journals and a plain study summary will be disseminated to people receiving mental health care, groups and forum that the project public members are connected, practitioners and commissioners. An abstract will be prepared for academic conferences such as the Society for Academic Primary Care Annual Conference.

For peer review only

For peer review only - http://bmjopen.bmj.com/site/about/guidelines.xhtml

AUTHORS' CONTRIBUTIONS

GY and YF conceived the study idea and design. GY drafted the initial manuscript, and GY, YF, and DC reviewed the manuscript and provided input to the final version.

FUNDING STATEMENT

This work is funded by the National Institute of Health and Care Research (NIHR) [Three Research Schools (MHF018)] and also supported by the NIHR [Applied Research Collaboration North East and North Cumbria (NIHR200173)]. The views expressed are those of the authors and not necessarily those of the NIHR or the Department of Health and Social Care.

COMPETING INTERESTS STATEMENT

The authors declare that they have no competing interests.

For peer review only

For peer review only - http://bmjopen.bmj.com/site/about/guidelines.xhtml

REFERENCES

1. Moreno C, Wykes T, Galderisi S, et al. How mental health care should change as a consequence of the COVID-19 pandemic. *The Lancet Psychiatry* 2020;7(9):813-24. doi: 10.1016/S2215-0366(20)30307-2
2. Pierce M, Hope H, Ford T, et al. Mental health before and during the COVID-19 pandemic: a longitudinal probability sample survey of the UK population. *The Lancet Psychiatry* 2020;7(10):883-92.
3. Banks J, Xu X. The mental health effects of the first two months of lockdown during the COVID-19 pandemic in the UK. *Fiscal Studies* 2020;41(3):685-708.
4. O'Shea N. Covid-19 and the nation's mental health: Forecasting needs and risks in the UK. 2020
5. Halstead EJ, Sullivan EC, Dimitriou D. The Impact of COVID-19 on Sleep in Autistic Adults: Longitudinal Comparisons pre and During Lockdown. *Frontiers in psychiatry* 2021:1608.
6. Qiu J, Shen B, Zhao M, et al. A nationwide survey of psychological distress among Chinese people in the COVID-19 epidemic: implications and policy recommendations. *General psychiatry* 2020;33(2)
7. Xiang Y-T, Yang Y, Li W, et al. Timely mental health care for the 2019 novel coronavirus outbreak is urgently needed. *The lancet psychiatry* 2020;7(3):228-29.
8. Anderson G, Frank JW, Naylor CD, et al. Using socioeconomics to counter health disparities arising from the covid-19 pandemic. *Bmj* 2020;369
9. Bambra C, Riordan R, Ford J, et al. The COVID-19 pandemic and health inequalities. *J Epidemiol Community Health* 2020;74(11):964-68.
10. Alderwick H, Dixon J. The NHS long term plan: British Medical Journal Publishing Group, 2019.
11. Shore JH, Schneck CD, Mishkind MC. Telepsychiatry and the coronavirus disease 2019 pandemic—current and future outcomes of the rapid virtualization of psychiatric care. *JAMA psychiatry* 2020;77(12):1211-12.
12. Garritty C, Gartlehner G, Nussbaumer-Streit B, et al. Cochrane Rapid Reviews Methods Group offers evidence-informed guidance to conduct rapid reviews. *Journal of clinical epidemiology* 2021;130:13-22.
13. Tricco AC, Langlois E, Straus SE, et al. Rapid reviews to strengthen health policy and systems: a practical guide: World Health Organization 2017.
14. McIntosh HM, Calvert J, Macpherson KJ, et al. The healthcare improvement Scotland evidence note rapid review process: providing timely, reliable evidence to inform imperative decisions on healthcare. *JBI Evidence Implementation* 2016;14(2):95-101.
15. Tricco AC, Antony J, Zarin W, et al. A scoping review of rapid review methods. *BMC medicine* 2015;13(1):1-15.
16. Polisena J, Garritty C, Kamel C, et al. Rapid review programs to support health care and policy decision making: a descriptive analysis of processes and methods. *Systematic reviews* 2015;4(1):1-7.
17. Featherstone RM, Dryden DM, Foisy M, et al. Advancing knowledge of rapid reviews: an analysis of results, conclusions and recommendations from published review articles examining rapid reviews. *Systematic reviews* 2015;4(1):1-8.
18. Grant MJ, Booth A. A typology of reviews: an analysis of 14 review types and associated methodologies. *Health information & libraries journal* 2009;26(2):91-108.
19. Page MJ, McKenzie JE, Bossuyt PM, et al. Updating guidance for reporting systematic reviews: development of the PRISMA 2020 statement. *Journal of Clinical Epidemiology* 2021;134:103-12.
20. NHS. Mental health conditions: NHS; 2022 [Available from: https://www.nhs.uk/mental-health/conditions/ accessed 08 Feb 2022.
21. Reibling N, Ariaans M, Wendt C. Worlds of Healthcare: A Healthcare System Typology of OECD Countries. *Health Policy* 2019;123(7):611-20. doi: https://doi.org/10.1016/j.healthpol.2019.05.001

For peer review only - http://bmjopen.bmj.com/site/about/guidelines.xhtml

22. Brunier A, Drysdale C. COVID-19 disrupting mental health services in most countries, WHO survey. *World Heal Organ* 2020
23. Byrne A, Barber R, Lim CH. Impact of the COVID-19 pandemic–a mental health service perspective. *Progress in Neurology and Psychiatry* 2021;25(2):27-33b.
24. EndNote [program]. EndNote X9 version. Philadelphia, PA: Clarivate, 2013.
25. Campbell M, Katikireddi SV, Sowden A, et al. Improving Conduct and Reporting of Narrative Synthesis of Quantitative Data (ICONS-Quant): protocol for a mixed methods study to develop a reporting guideline. *BMJ open* 2018;8(2):e020064.
26. Popay J, Roberts H, Sowden A, et al. Guidance on the conduct of narrative synthesis in systematic reviews. *A product from the ESRC methods programme Version* 2006;1(1):b92.
27. Popay J, Roberts H, Sowden A, et al. Guidance on the conduct of narrative synthesis in systematic reviews. *A product from the ESRC methods programme Version* 2006;1:b92.
28. Campbell M, McKenzie JE, Sowden A, et al. Synthesis without meta-analysis (SWiM) in systematic reviews: reporting guideline. *bmj* 2020;368

For peer review only

For peer review only - http://bmjopen.bmj.com/site/about/guidelines.xhtml

Mental health services in response to the COVID-19 pandemic in high-income countries: protocol for a rapid review

Appendix: Search Strategy

PubMed/Medline

	PubMed/Ovid Medline (1950 to date)
**COVID-19**	(2019 nCoV[tiab] OR 2019nCoV[tiab] OR corona virus[tiab] OR corona viruses[tiab] OR coronavirus[tiab] OR coronaviruses[tiab] OR COVID[tiab] OR COVID19[tiab] OR nCov 2019[tiab] OR SARS-CoV2[tiab] OR SARS CoV-2[tiab] OR SARSCoV2[tiab] OR SARSCoV-2[tiab] OR "COVID-19"[Mesh] OR "COVID-19 Testing"[Mesh] OR "COVID-19 Vaccines"[Mesh] OR "Coronavirus"[Mesh:NoExp] OR "Receptors, Coronavirus"[Mesh] OR "SARS-CoV-2"[Mesh] OR "Spike Glycoprotein, Coronavirus"[Mesh]) NOT ("animals"[mh] NOT "humans"[mh]) NOT (editorial[pt] OR newspaper article[pt]
**MENTAL HEALTH**	1. EATING DISORDERS/ or ANOREXIA NERVOSA/ or BINGE-EATING DISORDER/ or BULIMIA NERVOSA/ or FEMALE ATHLETE TRIAD SYNDROME/ or PICA/<br>2. HYPERPHAGIA/ or BULIMIA/<br>3. SELF-INJURIOUS BEHAVIOR/ or SELF MUTILATION/ or SUICIDE/ or SUICIDAL IDEATION/ or SUICIDE, ATTEMPTED/<br>4. MOOD DISORDERS/ or AFFECTIVE DISORDERS, PSYCHOTIC/ or BIPOLAR DISORDER/ or CYCLOTHYMIC DISORDER/ or DEPRESSIVE DISORDER/ or DEPRESSION, POSTPARTUM/ or DEPRESSIVE DISORDER, MAJOR/ or DEPRESSIVE DISORDER, TREATMENT-RESISTANT/ or DYSTHYMIC DISORDER/ or SEASONAL AFFECTIVE DISORDER/<br>5. NEUROTIC DISORDERS/<br>6. DEPRESSION/<br>7. ADJUSTMENT DISORDERS/<br>8. exp ANTIDEPRESSIVE AGENTS/<br>9. ANXIETY DISORDERS/ or AGORAPHOBIA/ or NEUROCIRCULATORY ASTHENIA/ or OBSESSIVE-COMPULSIVE DISORDER/ or OBSESSIVE HOARDING/ or PANIC DISORDER/ or PHOBIC DISORDERS/ or STRESS DISORDERS, TRAUMATIC/ or COMBAT DISORDERS/ or STRESS DISORDERS, POST-TRAUMATIC/ or STRESS DISORDERS, TRAUMATIC, ACUTE/<br>10. ANXIETY/ or ANXIETY, CASTRATION/ or KORO/<br>11. ANXIETY, SEPARATION/<br>12. PANIC/<br>13. exp ANTI-ANXIETY AGENTS/<br>14. SOMATOFORM DISORDERS/ or BODY DYSMORPHIC DISORDERS/ or CONVERSION DISORDER/ or HYPOCHONDRIASIS/ or NEURASTHENIA/<br>15. HYSTERIA/<br>16. MUNCHAUSEN SYNDROME BY PROXY/ or MUNCHAUSEN SYNDROME/<br>17. FATIGUE SYNDROME, CHRONIC/<br>18. OBSESSIVE BEHAVIOR/<br>19. COMPULSIVE BEHAVIOR/ or BEHAVIOR, ADDICTIVE/

For peer review only

	20. IMPULSE CONTROL DISORDERS/ or FIRESETTING BEHAVIOR/ or GAMBLING/ or TRICHOTILLOMANIA/<br>21. STRESS, PSYCHOLOGICAL/ or BURNOUT, PROFESSIONAL/<br>22. SEXUAL DYSFUNCTIONS, PSYCHOLOGICAL/ or VAGINISMUS/<br>23. ANHEDONIA/<br>24. AFFECTIVE SYMPTOMS/<br>25. *MENTAL DISORDERS/<br>26. (eating disorder* or anorexia nervosa or bulimi* or binge eat* or (self adj (injur* or mutilat*)) or suicide* or suicidal or parasuicid* or mood disorder* or affective disorder* or bipolar i or bipolar ii or (bipolar and (affective or disorder*)) or mania or manic or cyclothymic* or depression or depressive or dysthymi* or neurotic or neurosis or adjustment disorder* or antidepress* or anxiety disorder* or agoraphobia or obsess* or compulsi* or panic or phobi* or ptsd or posttrauma* or post trauma* or combat or somatoform or somati#ation or medical* unexplained or body dysmorphi* or conversion disorder or hypochondria* or neurastheni* or hysteria or munchausen or chronic fatigue* or gambling or trichotillomania or vaginismus or anhedoni* or affective symptoms or mental disorder* or mental health).ti.<br>27. or/1-26
**LOW AND MIDDLE INCOME COUNTRIES**	(afghanistan OR albania OR algeria OR american samoa OR angola OR "antigua and barbuda" OR antigua OR barbuda OR argentina OR armenia OR armenian OR aruba OR azerbaijan OR bahrain OR bangladesh OR barbados OR republic of belarus OR belarus OR byelarus OR belORussia OR byelORussian OR belize OR british honduras OR benin OR dahomey OR bhutan OR bolivia OR "bosnia and herzegovina" OR bosnia OR herzegovina OR botswana OR bechuanaland OR brazil OR brasil OR bulgaria OR burkina faso OR burkina fasso OR upper volta OR burundi OR urundi OR cabo verde OR cape verde OR cambodia OR kampuchea OR khmer republic OR cameroon OR cameron OR cameroun OR central african republic OR ubangi shari OR chad OR chile OR china OR colombia OR comORos OR comORo islands OR iles comORes OR mayotte OR democratic republic of the congo OR democratic republic congo OR congo OR zaire OR costa rica OR "cote d'ivoire" OR "cote d' ivoire" OR cote divoire OR cote d ivoire OR ivORy coast OR croatia OR cuba OR cyprus OR czech republic OR czechoslovakia OR djibouti OR french somaliland OR dominica OR dominican republic OR ecuadOR OR egypt OR united arab republic OR el salvadOR OR equatORial guinea OR spanish guinea OR eritrea OR estonia OR eswatini OR swaziland OR ethiopia OR fiji OR gabon OR gabonese republic OR gambia OR "geORgia (republic)" OR geORgian OR ghana OR gold coast OR gibraltar OR greece OR grenada OR guam OR guatemala OR guinea OR guinea bissau OR guyana OR british guiana OR haiti OR hispaniola OR honduras OR hungary OR india OR indonesia OR timOR OR iran OR iraq OR isle of man OR jamaica OR jORdan OR kazakhstan OR kazakh OR kenya OR "democratic people's republic of kORea" OR republic of kORea OR nORth kORea OR south kORea OR kORea OR kosovo OR kyrgyzstan OR kirghizia OR kirgizstan OR kyrgyz republic OR kirghiz OR laos OR lao pdr

For peer review only

	OR "lao people's democratic republic" OR latvia OR lebanon OR lebanese republic OR lesotho OR basutoland OR liberia OR libya OR libyan arab jamahiriya OR lithuania OR macau OR macao OR republic of nORth macedonia OR macedonia OR madagascar OR malagasy republic OR malawi OR nyasaland OR malaysia OR malay federation OR malaya federation OR maldives OR indian ocean islands OR indian ocean OR mali OR malta OR micronesia OR federated states of micronesia OR kiribati OR marshall islands OR nauru OR nORthern mariana islands OR palau OR tuvalu OR mauritania OR mauritius OR mexico OR moldova OR moldovian OR mongolia OR montenegro OR mORocco OR ifni OR mozambique OR pORtuguese east africa OR myanmar OR burma OR namibia OR nepal OR netherlands antilles OR nicaragua OR niger OR nigeria OR oman OR muscat OR pakistan OR panama OR papua new guinea OR new guinea OR paraguay OR peru OR philippines OR philipines OR phillipines OR phillippines OR poland OR "polish people's republic" OR pORtugal OR pORtuguese republic OR puerto rico OR romania OR russia OR russian federation OR ussr OR soviet union OR union of soviet socialist republics OR rwanda OR ruanda OR samoa OR pacific islands OR polynesia OR samoan islands OR navigatOR island OR navigatOR islands OR "sao tome and principe" OR saudi arabia OR senegal OR serbia OR seychelles OR sierra leone OR slovakia OR slovak republic OR slovenia OR melanesia OR solomon island OR solomon islands OR nORfolk island OR nORfolk islands OR somalia OR south africa OR south sudan OR sri lanka OR ceylon OR "saint kitts and nevis" OR "st. kitts and nevis" OR saint lucia OR "st. lucia" OR "saint vincent and the grenadines" OR saint vincent OR "st. vincent" OR grenadines OR sudan OR suriname OR surinam OR dutch guiana OR netherlands guiana OR syria OR syrian arab republic OR tajikistan OR tadjikistan OR tadzhikistan OR tadzhik OR tanzania OR tanganyika OR thailand OR siam OR timOR leste OR east timOR OR togo OR togolese republic OR tonga OR "trinidad and tobago" OR trinidad OR tobago OR tunisia OR turkey OR turkmenistan OR turkmen OR uganda OR ukraine OR uruguay OR uzbekistan OR uzbek OR vanuatu OR new hebrides OR venezuela OR vietnam OR viet nam OR middle east OR west bank OR gaza OR palestine OR yemen OR yugoslavia OR zambia OR zimbabwe OR nORthern rhodesia OR global south OR africa south of the sahara OR sub-saharan africa OR subsaharan africa OR africa, central OR central africa OR africa, nORthern OR nORth africa OR nORthern africa OR magreb OR maghrib OR sahara OR africa, southern OR southern africa OR africa, eastern OR east africa OR eastern africa OR africa, western OR west africa OR western africa OR west indies OR indian ocean islands OR caribbean OR central america OR latin america OR "south and central america" OR south america OR asia, central OR central asia OR asia, nORthern OR nORth asia OR nORthern asia OR asia, southeastern OR southeastern asia OR south eastern asia OR southeast asia OR south east asia OR asia, western OR western asia OR europe, eastern OR east europe OR eastern europe OR developing country OR developing countries OR developing nation? OR developing population? OR developing wORld OR less developed countr* OR less developed nation? OR less developed population? OR less developed wORld OR

	lesser developed countr* OR lesser developed nation? OR lesser developed population? OR lesser developed wORld OR under developed countr* OR under developed nation? OR under developed population? OR under developed wORld OR underdeveloped countr* OR underdeveloped nation? OR underdeveloped population? OR underdeveloped wORld OR middle income countr* OR middle income nation? OR middle income population? OR low income countr* OR low income nation? OR low income population? OR lower income countr* OR lower income nation? OR lower income population? OR underserved countr* OR underserved nation? OR underserved population? OR underserved wORld OR under served countr* OR under served nation? OR under served population? OR under served wORld OR deprived countr* OR deprived nation? OR deprived population? OR deprived wORld OR poOR countr* OR poOR nation? OR poOR population? OR poOR wORld OR poORer countr* OR poORer nation? OR poORer population? OR poORer wORld OR developing econom* OR less developed econom* OR lesser developed econom* OR under developed econom* OR underdeveloped econom* OR middle income econom* OR low income econom* OR lower income econom* OR low gdp OR low gnp OR low gross domestic OR low gross national OR lower gdp OR lower gnp OR lower gross domestic OR lower gross national OR lmic OR lmics OR third wORld OR lami countr* OR transitional countr* OR emerging economies OR emerging nation?).ti,ab,sh,kf.
**Notes**	[pt=publication type; ab=abstract; ti=title; fs=floating subheading; sh=subject heading; mp=title, original title, abstract, name of substance word, subject heading word, unique identifier]

For peer review only

PRISMA-P (Preferred Reporting Items for Systematic review and Meta-Analysis Protocols) 2015 checklist: recommended items to address in a systematic review protocol*

Section and topic	Item No	Checklist item	Line and Page number(s)
**ADMINISTRATIVE INFORMATION**			
Title:			
Identification	1a	Identify the report as a protocol of a systematic review	Page 1
Update	1b	If the protocol is for an update of a previous systematic review, identify as such	NA
Registration	2	If registered, provide the name of the registry (such as PROSPERO) and registration number	Page 5
Authors:			
Contact	3a	Provide name, institutional affiliation, e-mail address of all protocol authors; provide physical mailing address of corresponding author	Page 1
Contributions	3b	Describe contributions of protocol authors and identify the guarantor of the review	Page 8
Amendments	4	If the protocol represents an amendment of a previously completed or published protocol, identify as such and list changes; otherwise, state plan for documenting important protocol amendments	NA
Support:			
Sources	5a	Indicate sources of financial or other support for the review	Page 8
Sponsor	5b	Provide name for the review funder and/or sponsor	Page 8
Role of sponsor or funder	5c	Describe roles of funder(s), sponsor(s), and/or institution(s), if any, in developing the protocol	NA
**INTRODUCTION**			
Rationale	6	Describe the rationale for the review in the context of what is already known	Page 4
Objectives	7	Provide an explicit statement of the question(s) the review will address with reference to participants, interventions, comparators, and outcomes (PICO)	NA
**METHODS**			
Eligibility criteria	8	Specify the study characteristics (such as PICO, study design, setting, time frame) and report characteristics (such as years considered, language, publication status) to be used as criteria for eligibility for the review	Page 5
Information sources	9	Describe all intended information sources (such as electronic databases, contact with study authors, trial registers or other grey literature sources) with planned dates of coverage	Page 5
Search strategy	10	Present draft of search strategy to be used for at least one electronic database, including planned limits, such that it could be	Supplementary material

For peer review only

		repeated	
Study records:			
Data management	11a	Describe the mechanism(s) that will be used to manage records and data throughout the review	Page 5
Selection process	11b	State the process that will be used for selecting studies (such as two independent reviewers) through each phase of the review (that is, screening, eligibility and inclusion in meta-analysis)	Page 5
Data collection process	11c	Describe planned method of extracting data from reports (such as piloting forms, done independently, in duplicate), any processes for obtaining and confirming data from investigators	Page 6
Data items	12	List and define all variables for which data will be sought (such as PICO items, funding sources), any pre-planned data assumptions and simplifications	Page 6
Outcomes and prioritization	13	List and define all outcomes for which data will be sought, including prioritization of main and additional outcomes, with rationale	Page 5
Risk of bias in individual studies	14	Describe anticipated methods for assessing risk of bias of individual studies, including whether this will be done at the outcome or study level, or both; state how this information will be used in data synthesis	Page 6
Data synthesis	15a	Describe criteria under which study data will be quantitatively synthesised	Page 5
	15b	If data are appropriate for quantitative synthesis, describe planned summary measures, methods of handling data and methods of combining data from studies, including any planned exploration of consistency (such as I^2, Kendall's τ)	NA
	15c	Describe any proposed additional analyses (such as sensitivity or subgroup analyses, meta-regression)	Page 6
	15d	If quantitative synthesis is not appropriate, describe the type of summary planned	Page 6
Meta-bias(es)	16	Specify any planned assessment of meta-bias(es) (such as publication bias across studies, selective reporting within studies)	NA
Confidence in cumulative evidence	17	Describe how the strength of the body of evidence will be assessed (such as GRADE)	NA

*** It is strongly recommended that this checklist be read in conjunction with the PRISMA-P Explanation and Elaboration (cite when available) for important clarification on the items. Amendments to a review protocol should be tracked and dated. The copyright for PRISMA-P (including checklist) is held by the PRISMA-P Group and is distributed under a Creative Commons Attribution Licence 4.0.**

From: Shamseer L, Moher D, Clarke M, Ghersi D, Liberati A, Petticrew M, Shekelle P, Stewart L, PRISMA-P Group. Preferred reporting items for systematic review and meta-analysis protocols (PRISMA-P) 2015: elaboration and explanation. BMJ. 2015 Jan 2;349(jan02 1):g7647.

For peer review only
